# Exploring a new topological insulator in β-BiAs oxide†

**DOI:** 10.1039/d5ra01911g

**Published:** 2025-04-28

**Authors:** Tamiru Teshome

**Affiliations:** a Nanotechnology Center of Excellence, Addis Ababa Science and Technology University, College of Natural and Applied Sciences, Department of Mathematics, Physics and Statistics P. O. Box 16417 Addis Ababa Ethiopia tamiru.teshome@aastu.edu.et +251966253809

## Abstract

The scarcity of suitable quantum spin Hall (QSH) insulators with a significant bulk gap poses a major challenge to the widespread application of the QSH effect. This study employs first-principles calculations to investigate the stability, electronic structure, and topological properties of a fully oxygenated bismuth arsenide system. Without the influence of spin–orbit coupling (SOC), the valence and conduction bands at the *Γ*-point exhibit a semimetallic nature. However, introducing SOC leads to a substantial 352 meV band gap, which allows operation at room temperature. The calculation of the topological invariant reveals 
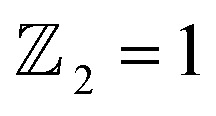
, and the presence of topologically protected edge states in a Dirac cone at the *Γ* point confirms the existence of a non-trivial topological state. The epitaxial growth of β-BiAsO_2_ on a SiO_2_ substrate maintains the band topology of β-BiAsO_2_, spin lock with SOC effect. Additionally, the fully oxidized surfaces of β-BiAsO_2_ are inherently resistant to surface oxidation and degradation, suggesting a promising approach for developing room-temperature topological quantum devices. These findings not only introduce new vitality into the 2D group-VA materials family and enrich the available candidate materials in this field but also highlight the potential of these 2D semiconductors as appealing ultrathin materials for future flexible electronics and optoelectronics devices.

## Abstract 2

This paper examines a BiAsO_2_ system *via* first-principles calculations, revealing a *E*_g_ =352 meV and , highlighting its potential for room-temperature quantum devices and flexible electronics.

## Graphical abstract

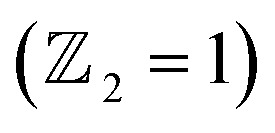

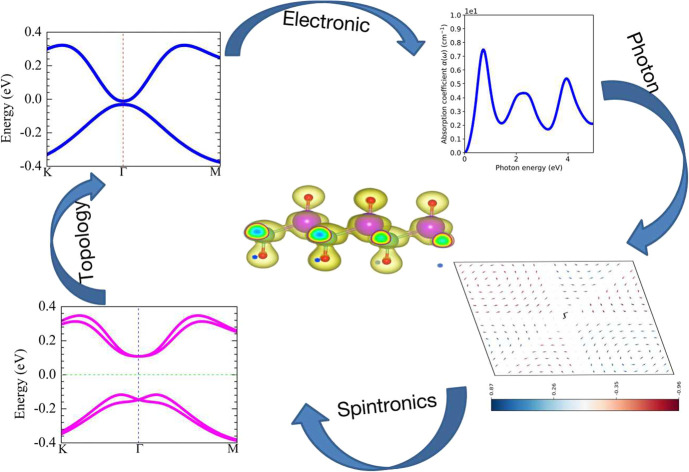

## 1 Introduction

The next generation of emerging devices is poised to leverage sophisticated semiconductors that exhibit high sensitivity to incident photons and efficient charge transport. This advancement is largely attributed to the unique properties of two-dimensional (2D) materials, which have recently gained significant attention as promising candidates for various applications. These materials are characterized by their exceptional transparency, mechanical flexibility, ultra-thin nature, high carrier mobility, and strong interaction with light, making them ideal for optoelectronic devices and next-generation electronics. The versatility of 2D materials has opened up new possibilities in fields such as photovoltaics, sensors, flexible electronics, and quantum technologies. Among the most notable materials in this context are the group VA elements. These materials have emerged as a significant area of research due to their large fundamental band gaps,^1–3^ which enhance their potential for use in semiconducting applications. The semiconducting behaviors of group VA 2D materials position them as viable candidates for optoelectronic devices, particularly when compared to other 2D materials such as group IVA (including silicene, graphene, germanene, and stanene) and group IIIA (borophene). For instance, despite its high carrier mobility and mechanical strength, graphene lacks a natural band gap, limiting its utility in semiconductor devices. In contrast, group VA materials offer tunable band gaps and robust electronic properties, making them more suitable for practical applications in electronics and photonics.^4^

The exploration of topological insulators (TIs) has also gained momentum, particularly with the contributions of researchers like Kane and Mele, who first introduced the concept of the quantum spin Hall (QSH) insulator in graphene.^5^ Their groundbreaking work demonstrated that materials with strong spin–orbit coupling (SOC) could exhibit insulating behavior in the bulk while conducting electricity on their surfaces through topologically protected edge states. However, the spin–orbit coupling (SOC) in graphene is relatively weak, resulting in a very small band gap at the Dirac point, ∼10^−3^ meV. This limitation has spurred interest in other 2D materials with stronger SOC effects, such as Bi_2_Se_3_ and Sb_2_Te_3_, which exhibit larger band gaps and more robust topological properties.^6,7^ The emergence of band topology has opened new avenues in the study of topological quantum states, leading to the discovery of various exotic phases of matter, including Weyl semimetals,^8^ topological insulators (TIs), topological Dirac semimetals, and node-line semimetals.^9^ For example, Weyl semimetals host massless fermions known as Weyl fermions, which are characterized by their chiral nature and high mobility. These properties make them promising candidates for low-power electronics and quantum devices. Similarly, topological insulators, with their surface states protected by time-reversal symmetry,^5^ offer potential applications in fault-tolerant quantum computing and energy-efficient electronics. The integration of group VA 2D materials into the next generation of electronic devices holds great promise due to their favorable semiconducting properties and the potential for novel applications in optoelectronics and quantum technologies.^10^ The ongoing research into their unique characteristics and behaviors, including their electronic, optical, and thermal properties, will likely lead to significant advancements in the field. Moreover, developing scalable synthesis techniques and device fabrication methods will be crucial for translating these materials from the laboratory to real-world applications.^11^ As researchers uncover new insights into the fundamental properties of these materials, their potential to revolutionize technology across multiple domains becomes increasingly evident.

Two-dimensional (2D) topological insulators (TIs),^1–3^ particularly quantum spin Hall (QSH) insulators,^12–15^ represent a significant advancement in the field of condensed matter physics and materials science. These materials exhibit unique electronic properties due to their topologically protected edge states, which allow electrons to flow along the edges of the material without significant backscattering.^5^ This characteristic gives 2D-TIs a slight advantage over their three-dimensional (3D) counterparts, as the restriction of electron flow to 2D inherently limits backscattering and enhances conductivity. The robustness of these edge states against non-magnetic impurities and defects makes 2D-TIs highly promising for applications in low-power electronics, spintronics, and quantum computing. Several 2D materials have been theoretically predicted and experimentally investigated for their potential to exhibit topological insulator phases. Among these are silicene,^16^ germanene,^16^ phosphorene,^17^ arsenene,^18^ antimonene,^19^ and bismuthine.^6,20–24^ These materials are particularly intriguing because of their ability to undergo a topological phase transition from normal insulators to topological insulators under specific conditions, such as strain, electric fields, or chemical functionalization. For instance, silicene and germanene, which are the silicon and germanium analogs of graphene, have been shown to exhibit topological insulating behavior when subjected to external perturbations. Similarly, stanene, a tin-based 2D material, has been predicted to host a large-gap QSH state,^25–27^ making it a strong candidate for room-temperature applications.^22^ Bismuthine has also garnered significant attention due to its strong spin–orbit coupling (SOC) and potential for topological phase transitions. Hence, antimonene has been shown to possess a sizable band gap and robust topological properties, making it a promising material for next-generation electronic devices.^28^

Experimentally, the realization of 2D-TIs has been demonstrated in systems such as HgTe/CdTe and InAs/GaSb quantum wells.^29^ These systems have been extensively studied and confirmed to exhibit QSH insulator behavior at very low temperatures (below 10 K). The HgTe/CdTe quantum well, in particular, was the first experimentally verified 2D-TI, showcasing quantized edge conductance due to the presence of helical edge states. However, despite these successes, significant challenges remain. The small bulk band gaps of these materials, typically on the order of a few meV, limit their operational temperature range and make them unsuitable for ambient temperature applications.^30–32^ In addition, these materials with conventional semiconductor devices pose technical challenges as a result of differences in material properties and fabrication processes. The limitations of current 2D TIs have spurred research into alternative materials and strategies to achieve larger band gaps and higher operating temperatures. Moreover, strain, external electric fields, or chemical doping have been proposed to enhance the topological properties of existing 2D materials.^17,18,22^ Advances in material synthesis and characterization techniques, such as molecular beam epitaxy (MBE)^33^ and angle-resolved photoemission spectroscopy (ARPES),^34^ have also played a crucial role in the discovery and understanding of new 2D TIs.^6,35,36^ While challenges such as small band gaps and low operational temperatures remain. The discovery of new 2D TIs with robust topological properties and compatibility with existing technologies will be critical for realizing their full potential in practical applications.^37–44^ In addition, due to their strong SOC effect, bismuth and arsenic are full of electronic sources in buckled and planar hexagonal formations, such as bismuth-based systems, which are good materials for TIs. For example, Bi_2_Se_3_ and Bi_2_Te_3_ are well-known binary compounds known as strong TIs.^45,46^ Arsenene in the α and β-phases, on the other hand, were tuned from conventional insulators to topological insulators under strain modification and were found to be energetically stable at ambient temperature.^47–50^ As a result, finding a suitable 2D topological insulator with a wide band gap is an important research topic. Therefore, finding nontoxic and experimentally feasible QSH insulators is critical. Thus, radical functionalizations like methyl (–CH_3_), ethynyl(–C_2_H), and cyano(–CN) group have been proposed QSH effect.^32,39,51,52^ Based on first-principles calculations, BiAs oxide at room temperature provides a promising candidate for designing future electronic devices.

## 2 Computational details

The first principle calculations were implemented by the Vienna *ab initio* simulation package (VASP).^53,54^ The exchange–correlation term is described within the generalized gradient approximation (GGA) parameterized by the Perdew–Burke–Ernzerhof (PBE) functional.^55^ A large vacuum space of 25 Å is set along the *c*-axis to avoid the interaction between layers caused by the periodic boundary condition. The kinetic-energy cutoff plane-wave functions are 450 eV, and all atom coordinates and lattice constants were optimized until the convergence of force on each atom less than 0.001 eV Å^−1^ and the total energy convergence threshold was set to be 10^−6^ eV. The *k*-mesh of 5 × 5 × 1 and 11 × 11 × 1 Monkhorst–Pack *k*-point grid was adopted for geometry optimization and self-consistent calculation, respectively.^54^ The effect of SOC being included in self-consistently in the electronic structure calculations.^56^ Beside SOC effect,^57^ the hybrid HSE06 functional addresses some of the limitations of the PBE functional in predicting band structures by providing better estimates of the electronic band gaps.^58^ Phonon calculations are performed using the PHONOPY code^56^ combined with the density functional perturbation theory (DFPT) method in VASP, and *ab initio* molecular dynamics (MD) simulations^59^ were performed to verify stabilities at 300 K. Berry phase and Wannier loop functions characterized by topological invariants 
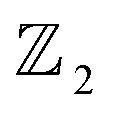
 package.^60–63^

## 3 Results and discussion

We previously explored the electronic structures of two-dimensional (2D) bismuth arsenide (BiAs) polymorphs. Our investigations revealed that among these new materials, the β-BiAs adopts a stable structure. Notably, we observed a significant phase transition in β-BiAs, shifting from a normal insulator to a topological insulator as we applied a tensile strain of 12%. This transition occurred without and with spin–orbit coupling (SOC) effects.^64^ This paper presents oxygenated β-BiAs as a novel class of strain-effect-free 2D topological insulators. The addition of oxygen to the β-BiAs structure improve its electrical characteristics, opening up new research directions in the field of topological materials. The oxygenated form of β-BiAs, to evaluate its topological properties, electronic band structure, and stability. This work not only contributes to our understanding of the behavior of β-BiAs but also paves the way for the development of novel materials that may exhibit robust topological states in practical applications. Bismuth is known for its high mobility of charge carriers, which facilitates efficient spin transport, an essential feature for spintronics applications that leverage both charge and spin degrees of freedom,^19^ and bismuthine.^6,20–24,65^ Its distinctive electronic properties make it suitable for use in magnetic tunnel junctions, thereby improving the performance of memory devices. Furthermore, adding bismuth to spin transistors can provide devices that make use of electron spin, which may lead to lower power consumption and faster speeds. Bi_2_As_3_ is one of the bismuth compounds that exhibits topological insulating properties. Bismuth's special qualities also make it a strong contender for quantum bits in quantum computing, where efficient spin state management is essential. The investigation of fully oxygenated β-BiAs is a promising development in the search for novel materials with topological characteristics that may result in important breakthroughs in electrical devices of the future. In the β-BiAs monolayer, each arsenic atom is covalently bonded to three neighboring bismuth atoms, forming sp^2^-like hybridized orbitals. This arrangement is complemented by the presence of weak π orbitals, which contribute to the overall electronic structure of the material. Due to this bonding configuration, nonbonding lone-pair electrons reside in the remaining orbitals of each arsenic atom. This unique electronic band structure characteristic as shown in Fig. S1(a)† renders the system particularly susceptible to forming dative bonds with other elements that possess strong electronegativity, such as oxygen and fluorine.

Given this propensity for bonding, the creation of stable oxygenated β-BiAs monolayers. These functionalized materials could exhibit significantly altered properties, opening up new possibilities for applications in various fields. In this study, focused on the effects of oxygenation in β-BiAs by examining three distinct structural configurations. These configurations include: (i) O–As–Bi as shown in Fig. S2(a)† an oxygen atom is bonded primarily to the arsenic atom, which influences the electronic properties as shown in Fig. S2(b and c)† and the stability of the monolayer. (ii) As–Bi–O as shown in Fig. S3(a)† the oxygen atom is attached to the bismuth atom, potentially altering the electronic interactions within the material and enhancing its reactivity or conductivity as shown in Fig. S3(b and c)† and (iii) O–Bi–As–O this configuration involves the incorporation of two oxygen atoms, which could lead to more pronounced changes in the electronic structure and the overall stability of the β-BiAsO_2_ monolayer as illustrated in Fig. S4(a–d).† By investigating these three structures O–As–Bi, As–Bi–O, and O–Bi–As–O to understand how the oxygen affects the electronic characteristics and stability of β-BiAs. This work could provide valuable insights into the design of new materials that leverage the unique properties of oxygen-functionalized β-BiAs, ultimately contributing to advancements in electronic devices and other applications that require materials with specific electronic and chemical properties.

**Fig. 1:**
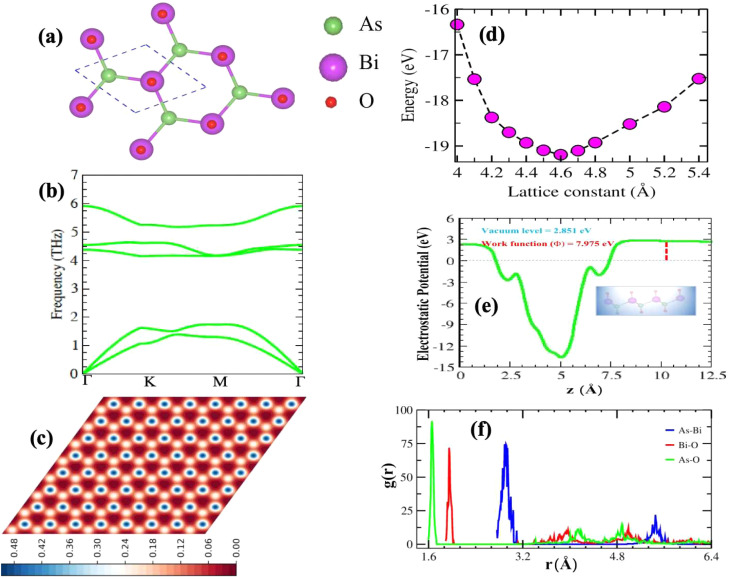
(a) The optimized structure of β-Bop view, (b) phonon dispersion spectrum, (c) Scanning Tunneling Microscope (STM) image, (d) energy fluctuation as a function of lattice parameter, (e) electrostatic potential, and (f) radial distribution function.

As illustrated in Fig. S4(a)† the optimized lattice structure of β-BiAsO_2_. This structure adopts a hexagonal configuration characteristic of oxygenated β-BiAs, which comprises four atoms per unit cell. The specific parameters of this lattice reveal important insights into its geometric and electronic properties. The buckling height of the structure is measured at 1.56 Å. The buckling height is significant because it influences the electronic properties and stability of the monolayer. The lattice constant is calculated as 4.605 Å, which defines the repeating unit of the crystal structure in the 2D plane. The Bi–As bond length is 2.94 Å, indicating a longer covalent interaction between the bismuth and arsenic atoms. Furthermore, the Bi–O bond length is calculated to be 1.95 Å, suggesting a robust interaction between the bismuth and oxygen atoms. This shorter bond length typically implies a stronger interaction, which could enhance the stability of the oxygenated structure and facilitate the formation of dative bonds with electronegative elements. The bond length of the As–O atoms is calculated to be 1.7 Å. This length reflects the nature of the bonding interactions within the structure and may influence how the β-BiAsO_2_ behaves under different conditions, such as temperature fluctuations or external pressures. Hence, the detailed analysis of the optimized lattice structure of β-BiAsO_2_ not only highlights the specific geometric parameters but also underscores the potential implications for its electronic properties as shown in Fig. S4(b–d)† and suitability for various applications in advanced materials and devices. Understanding these characteristics is vital for further exploration and optimization of oxygenated β-BiAs for practical uses in electronics, photonics, and beyond. The optimized structure of BiAsO_2_ reveal its hexagonal arrangement as shown in Fig. 1(a). The clear depiction of the bonding geometry indicates the presence of covalent interactions between Bi and As, as well as between Bi and O, which are essential for the material's robustness. As shown in Fig. 1(d) the energy fluctuation as a function of the lattice parameter provides insights into the stability of the β-BiAsO_2_ structure. The curve indicates that there is a specific lattice constant at which the energy is minimized, suggesting that this is the most stable configuration for β-BiAsO_2_ at 4.6 Å. Deviations from this optimal lattice parameter lead to increased energy, which can imply potential instability or unfavorable properties. The electrostatic potential as observed in Fig. 1(e) the variation in potential energy across the vacuum level, and the work function are around 2.851 eV and 7.975 eV, respectively. The presence of distinct potential wells can indicate regions of varying electronic density, which influence charge transport and reactivity. Analyzing the electrostatic potential profile helps in understanding how β-BiAsO_2_ interacts with external electric fields, which is particularly relevant for electronic and optoelectronic applications. The Scanning Tunneling Microscope (STM) image as shown in Fig. 1(c) provides a real-space visualization of the surface morphology of β-BiAsO_2_. The high-resolution image captures the atomic arrangement and allows for the identification of surfaces. The contrast in the STM image can reflect variations in local electronic states, which can further inform on the material's surface electronic properties. The formation energy can be used to estimate the structure's stability. The formation energy is calculated using the following expression:

1 *E*_f_ = *E*_T_ − *E*_BiAs_ − O_2_

where *E*_T_ and *E*_BiAs_ are the energies of β-BiAs systems with and without oxygen, and the energy of O_2_ molecule. The binding energy is calculated to be −1.75 eV per unit cell.

**Fig. 2:**
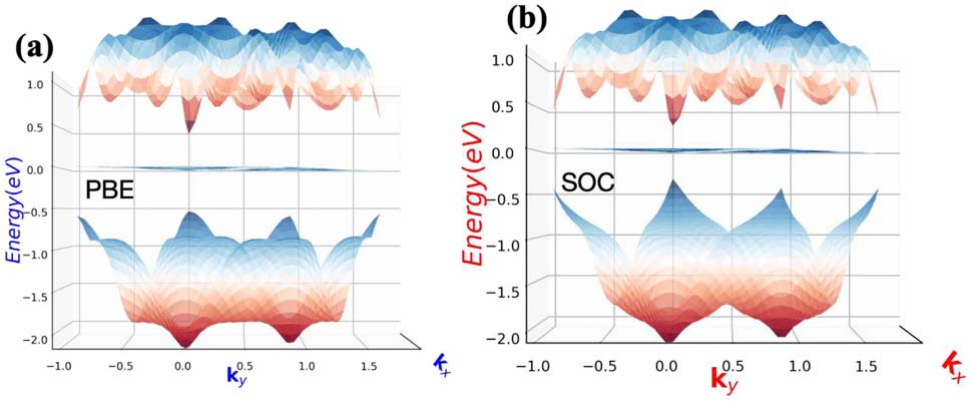
Electronic band structures of free standing β-BiAs (a) without and (b) with SOC, respectively.

**Fig. 3:**
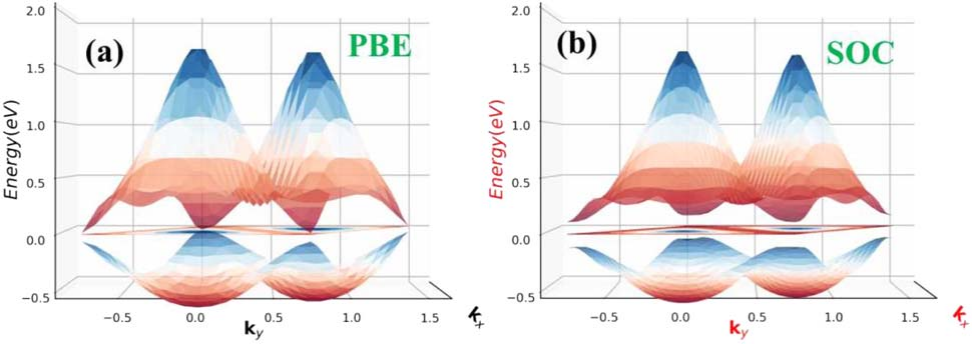
Surface functionalizing of β-BiAsO_2_ band structures (a) without SOC and (b) with SOC. The Fermi level set to be 0 eV.

The absences of imaginary frequencies show that β-BiAsO_2_ is dynamically stable as shown in Fig. 1(b). Additionally, molecular dynamics (MD) simulations were performed on the β-BiAsO_2_ for 10 ps with a time step of 1.0 fs at 300 K. The MD simulations indicate that these β-BiAsO_2_ were also thermally stable at room temperature. The radial distribution function (RDF) analysis for As–Bi, Bi–O, and As–O, bonds provides insights into the local structure and bonding characteristics of the material as shown in Fig. 1(f) peaks in the RDF correspond to specific bond lengths, indicating the presence of preferred distances between atoms for As–Bi, Bi–O, and As–O around 2.95 Å, 1.6 Å and 1.7 Å, respectively. This information is essential for understanding the interactions within the material and can help predict its mechanical, thermal, and electronic properties. The RDF can also reveal how these interactions change with different conditions, such as temperature or external pressure. What effect do these oxidations have on β-BiAsO_2_ electrical properties? To solve this problem, the electronic band structures of 2D β-BiAs oxides were calculated. The effect of oxygen functionalization on the electronic and structural properties of β-BiAs monolayers is illustrated through various Fig. 2 and 3. The band gap for free standing β-BiAs calculated at the Perdew–Burke–Ernzerhof (PBE) level is reported to be a notable 1.02 eV in the absence of spin–orbit coupling (SOC) effect. This value indicates that β-BiAs behaves as a semiconductor, with a significant energy gap that separates the valence and conduction bands as shown in Fig. 2(a and b) with and without SOC effect. When we compare this to the band structure of the β-BiAsO_2_ monolayer, as shown in Fig. 3(a and b), the band structure reveals gapless semiconductor behavior, with the valence band maximum and conduction band minimum both located at the *Γ* point. This indicates that the introduction of oxygen functionalization effectively alters the electronic properties of the material, suggesting a transformation from a bandgap semiconductor to a topological insulator with gapless characteristics. For the structures As–Bi–O and Bi–As–O, shown in Fig. S2(c) and S3(c),† the electronic band gaps are significantly reduced to approximately 0.003 eV and 0.006 eV, respectively. These extremely small band gaps suggest that both functionalized structures approach a metallic state, where the distinction between the valence and conduction bands is minimal. In contrast to the various derivatives of β-BiAsX (X = Br_2_, CH_3_, CN, Cl_2_, F_2_, H_2_, I_2_, and NH_2_), as shown in Table 1 and Fig. S5, S6,† the electronic properties of these materials reveal significant differences. For the PBE calculations, the semiconductor band gaps were observed to lie between 0.45 eV and 1.75 eV as illustrated in Fig. S7–S10.† This range suggests that the materials can effectively facilitate electronic transitions at relatively low energy levels, making them suitable for applications in optoelectronic and photo-detection, where lower energy photons are of interest. In the HSE06 for calculations the band gap expands to a range of 0.63 eV to 2.08 eV. In contrast of the functional β-BiAsX (X = Br_2_, CH_3_, CN, Cl_2_, F_2_, H_2_, I_2_, and NH_2_) HSE06 bandgap around 0.009 eV as shown in Fig. S13(a and b).† This broader range indicates a more refined assessment of the electronic structure, reflecting the influence of hybridization and the exchange–correlation effects captured by the HSE06 method. The higher band gap values suggest improved electronic properties, which could enhance the material's performance in electronic devices where a wider band gap is beneficial. The variation in band gap values underscores the potential for tailoring these materials for specific applications through careful selection of their chemical composition and the computational techniques employed to analyze their properties.

**Table 1:** The radical functionalization of BiAsX (X = Br_2_, Cl_2_, CH_3_, CN, F_2_, H_2_, I_2_, NH_2_ and O_2_), lattice constant (Å), bond length (Å), buckling height (Å), Bader charge analysis, and band gaps (PBE, SOC and HSE06) *E*_g_ (eV)

Model	*a* = *b*	*d*_Bi–X_	*d*_As–X_	Δ*h*	Δ*Q*	PBE	SOC	HSE06
Br_2_	4.60	2.67	2.85	1.18	0.79	1.04	1.06	1.26
Cl_2_	4.56	2.66	2.84	1.17	0.78	1.01	1.04	1.25
CH_3_	4.60	2.62	2.81	1.13	0.79	1.02	1.04	1.24
F_2_	4.59	2.58	2.72	1.14	0.81	1.08	1.10	1.33
H_2_	4.51	2.81	2.87	0.09	0.77	1.01	1.03	1.25
I_2_	4.59	2.81	2.87	0.09	0.79	1.01	1.03	1.27
NH_2_	4.60	2.81	2.79	0.02	0.78	1.01	1.03	1.24
O_2_	4.605	2.810	2.791	1.560	0.772	0.004	0.352	0.009

Furthermore, the Dirac cones observed in these structures indicate that the VBM and CBM are closely aligned, occurring at the *Γ*-point for Bi–As–O and As–Bi–O as shown in Fig. S2(c) and S3(c).† The oxidation not only reduces the band gap but also leads to the emergence of Dirac-like states, suggesting the potential for new electronic properties and applications for these functionalized materials *E*_g_ = 352 meV with SOC as shown in Fig. 3(b) and S4(c).† This highlights the importance of oxygen functionalization in tuning the electronic characteristics of β-BiAs monolayers, indicating a pathway toward designing materials with desired electronic properties for future applications in electronic devices and topological insulators.

**Fig. 4:**
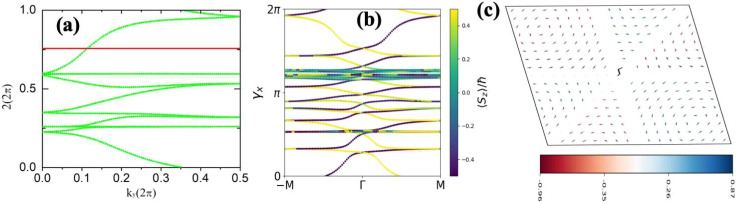
(a) The Wilson loop (Wannier Charge Center) along *k*_*y*_ for β-BiAsO_2_ yielding 
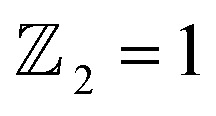
, (b) Berry phase spectrum and (c) spin texture of β-BiAsO_2_.

The topological properties and spin characteristics of β-BiAsO_2_ as shown in Fig. 4(a–c), includes three primary components: the calculation of the Wilson loop, the Berry phase spectrum, and the spin texture of β-BiAsO_2_. The calculation of the Wilson loop along the *k*_*y*_ direction provides essential information about the topological nature of β-BiAsO_2_ as shown in Fig. 4(a). The p-orbitals close to the Fermi level separate into two categories: the p_*x*_ and p_*y*_ orbitals, and the p_*z*_ orbital. By projecting the bands of the p_*x*_, p_*y*_, and p_*z*_ orbitals onto various atomic orbitals, it was observed that the energy spectrum of β-BiAsO_2_ near the Fermi level is primarily influenced by the p_*x*_ and p_*y*_ orbitals. In this context, the Wilson loop is used to determine the position of the Wannier charge centers, which are indicative of the material's topological invariants. The Wannier function (*W*_*n*_(***r***)) is obtained by Fourier transforming the Bloch states:2
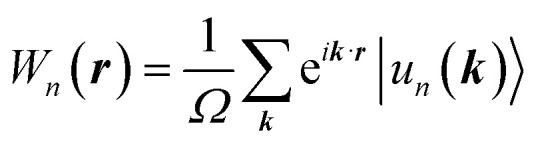
where *Ω* is the volume of the Brillouin zone, |*u*_*n*_(***k***)〉 are the periodic parts of the Bloch functions. The topological invariant can then be computed as:3
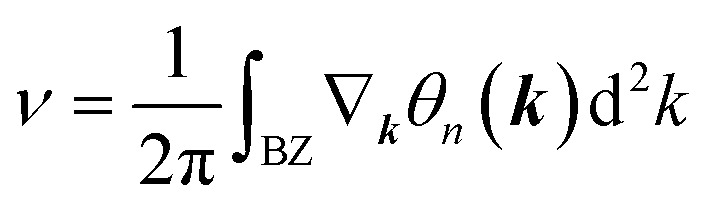
where *θ*_*n*_(***k***) is the phase of the overlap between adjacent Wannier functions.^60–63^ The result yielding 
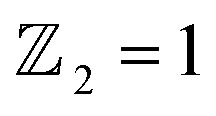
 confirms that β-BiAsO_2_ is topological insulator. The value of 
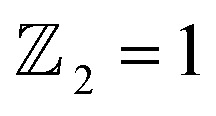
 signifies that the material has protected edge states. These edge states arise due to the non-trivial topology of the band structure, making the material robust against certain types of perturbations. The Berry phase spectrum provides additional insight into the electronic structure of β-BiAsO_2_ as shown in Fig. 4(b). This spectrum illustrates how the Berry curvature varies across the Brillouin zone. The peaks and troughs in the spectrum correspond to different electronic bands and highlight regions where the Berry curvature is significant. The Berry curvature is directly related to the topological properties of the material and can influence phenomena such as the anomalous Hall effect. The visualization of the Berry phase spectrum enables a deeper understanding of the interplay between the electronic bands and the topological features of the material, further corroborating its classification as a topological insulator. The *k*-space Berry phases can be applied to extract topological properties of solids. For an isolated band, the Berry phase is calculated4
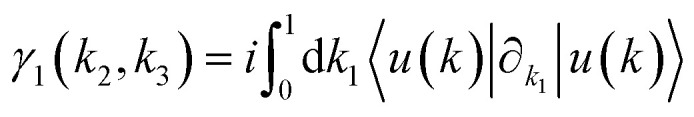
where *k*_*i*_ is the component of crystal momentum corresponding to ***b***_*i*_ in reduced coordinates. The Berry phase is only gauge invariant modulo 2π and since *γ*_1_(*k*_2_, *k*_3_) = *γ*_1_(*k*_2_ + 1, *k*_3_) = *γ*_1_(*k*_2_, *k*_3_ + 1) modulo 2π, it can be count how many multiples of the 2π that *γ*_1_ changes while *k*_2_ of *k*_3_ is adiabatically cycled through the Brillouin zone. This is the Chern number, which gives rise to a topological 
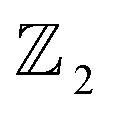
 classification of all 2D insulators. For multiple valence bands the situation is slightly more complicated and one has to introduce the notion of parallel transport to obtain the Berry phases of individual bands.^62^ The spin texture illustrated in Fig. 4(c) represents the spatial distribution of the spin polarization across the surface of β-BiAsO_2_. The arrows indicate the orientation of the spin, while the color gradient reflects the magnitude of the spin polarization. This visualization is crucial for understanding the spin properties of the material. The presence of a well-defined spin texture suggests that the spins are coupled to the electronic states in a way that could enable spin-polarized currents, potentially leading to devices that utilize both charge and spin for enhanced performance. Moreover, effectively as shown in Fig. 4(a–c) the topological and spin properties of β-BiAsO_2_. The calculations of the Wilson loop confirm its status as a topological insulator, while the Berry phase spectrum and spin texture provide crucial insights into its electronic behavior and spin dynamics. Together, these elements underscore the potential of β-BiAsO_2_ for advanced applications in electronic and spintronic devices.

**Fig. 5:**
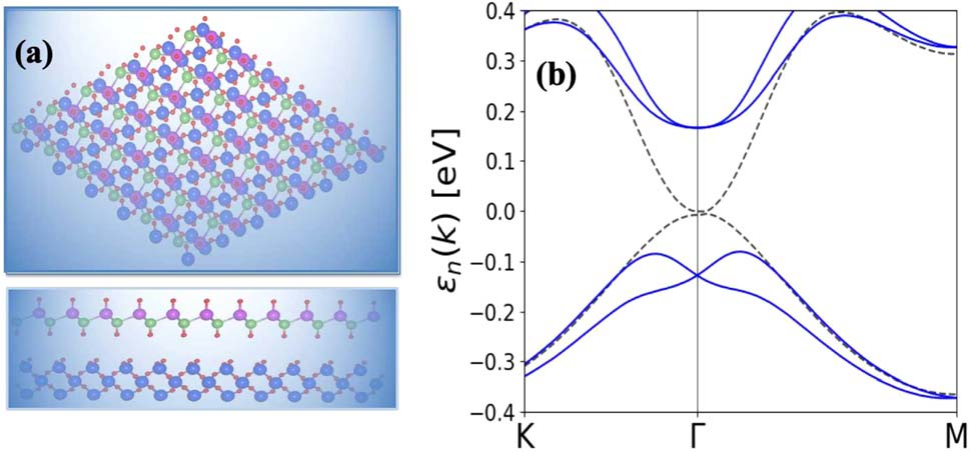
(a) Side and top view of epitaxial growth β-BiAsO_2_ of topological insulator and (b) SiO_2_ substrate without and with SOC. The Dirac state of β-BiAsO_2_ edge spins polarization.

As shown in Fig. 5(a and b) the epitaxial growth of β-BiAsO_2_ as a topological insulator and its electronic properties, particularly to spin polarization. The side and top views of the structure β-BiAsO_2_ show its layered arrangement, which is crucial to understanding its growth on a SiO_2_ substrate, as shown in Fig. 5(a). The epitaxial growth process significantly affects the electronic properties of the β-BiAsO_2_ material, including its band structure and spin polarization. The structure of the band as shown in Fig. 5(b) provides crucial information on the electronic states of β-BiAsO_2_, both without and with spin orbit coupling (SOC). The presence of the Dirac states is evident, which are characteristic of topological insulators. These states are marked by a crossing of the conduction and valence bands at the Dirac point, indicating a zero band gap at *Γ* point. In the absence of SOC, the band structure may display a simpler electronic configuration. However, the inclusion of SOC introduces significant modifications, leading to a rich spin texture. The band structure plot suggest that the spin polarization is nontrivial, with the Dirac edge states exhibiting polarization characteristics. This polarization can lead to the generation of spin-polarized currents, which are invaluable for developing next-generation devices that utilize both charge and spin for information processing. Hence, this figure effectively illustrates the epitaxial growth of β-BiAsO_2_ on a SiO_2_ substrate and highlights its electronic properties through the band structure analysis. The insights gained from the structural and electronic characteristics underscore the potential of β-BiAsO_2_ as a topological insulator, particularly in applications that leverage its unique spin polarization features. Understanding these aspects can guide further research and development in the field of spintronics and novel electronic materials. The lattice mismatch between the β-BiAsO_2_ and SiO_2_ is measured to be just 0.07 Å, which is quite minimal and indicative of a well-matched interface. This small mismatch, combined with an interlayer distance of 4.102 Å, facilitates the formation of a stable van der Waals (vdW) heterostructure. Such stability is essential for ensuring the integrity and performance of the material system during practical applications. The binding energy of the heterostructure has been calculated to be −0.032 eV per unit cell. This negative value suggests that the interactions holding the layers together are relatively weak and are primarily due to dispersion forces, which are characteristic of vdW interactions. Such weak binding can be beneficial in applications where flexibility and tunability of the layers are desired, allowing for easy manipulation of the heterostructure properties without significant energy penalties. The band structure of the β-BiAsO_2_/SiO_2_ heterostructure shows remarkable stability, remaining largely unperturbed regardless of the inclusion of spin–orbit coupling (SOC), as shown in Fig. 5(b). The electronic states of the heterostructure are robust against changes in the electronic environment. Furthermore, Bader charge analysis confirms that there is no significant charge transfer occurring between the β-BiAsO_2_ and the SiO_2_ layers. This lack of charge transfer is critical, as it suggests that the electronic properties of β-BiAsO_2_ remain intact when integrated with SiO_2_, allowing the material to retain its unique characteristics. The orbitals near the Fermi level are predominantly contributed by the bismuth and arsenic atoms, indicating that these elements play a crucial role in defining the electronic behavior of the heterostructure. Additionally, calculating the charge density difference further supports these findings, revealing the spatial distribution of charge within the heterostructure. Understanding these electronic interactions and charge distributions is vital for optimizing the performance of the β-BiAsO_2_/SiO_2_ system. Thus, a minimal lattice mismatch, weak binding interactions, and unperturbed electronic properties position this vdW heterostructure as a promising candidate for future technological advancements.

Recently, significant progress has been made in the synthesis of various 2D TIs.^39,48,66–72^ This expansion of materials showcases a promising diversity for potential applications in quantum computing, spintronics, and other advanced electronic devices. Among the recently synthesized 2D TIs, bismuthene,^19^ and bismuthine^6,20–24^ has demonstrated unique electronic properties that can be attributed to strong spin–orbit coupling (SOC), making it a robust candidate for further exploration. Similarly, plumbene^73^ and stanene^74^ have attracted attention for their potential to exhibit nontrivial topological behavior at room temperature, driven primarily by their structural characteristics and the presence of heavy elements with significant SOC. Furthermore, the β-BiAsO_2_/SiO_2_ heterostructure is a notable example in this emerging category of materials. Its topology remains stable and is not influenced by perturbations from the substrate on which it is grown. This stability is essential for practical applications, as it may alleviate issues related to substrate-induced instability that have affected previous materials under external influences. A key aspect of topological insulators is their band gap characteristics. Generally, many heavy metal-based 2D TIs exhibit significant bulk gaps, often exceeding 0.1 eV, due to their strong spin–orbit coupling (SOC) interactions. On the other hand, some lighter elements, while showing promise as TIs, often exhibit smaller bulk gaps. For instance, graphene displays a gap as small as 10^−3^ meV,^5^ while silicene's gap ranges from 1.55 to 2.9 meV,^16^ and germanene's gap is between 23.9 and 108 meV.^17^ Conversely, arsenene is noted to have a more significant gap of approximately 696 meV,^18^ and few-layered black phosphorus is reported at 5 meV.^17^ Furthermore, arsenene oxide has garnered attention due to its somewhat larger gap of 232 meV,^49,50^ hinting at the effects of oxidation^37,75,76^ on the electronic configuration of the base arsenene structure.^70,71,77–82^ Notably, the newly predicted 2D β-BiAsO_2_ binary compound appears to have a sizable gap up to 352 meV, indicative of its substantial potential as a robust nontrivial topological insulator.

In general, research on 2D topological insulators (TIs), particularly those composed of heavy metals with strong spin–orbit coupling (SOC), has facilitated improvements in material properties that are applicable in practice. The synthesis of materials such as β-BiAsO_2_ and arsenene oxide not only underscores the significance of bulk gap size in shaping TI behavior but also highlights the potential to investigate new materials with improved topological features. As we explore the relationships among material composition, structural properties, and substrate interactions, the field of 2D TIs is likely to continue evolving, creating further opportunities for innovation in advanced electronics and spintronic applications. Developing comprehensive theoretical and experimental frameworks to understand these complex materials will be crucial for guiding future research and designing next-generation topological insulator devices. The expanding catalog of 2D TIs represents an exciting frontier in materials science, driven by both fundamental research interests and practical technological goals. Progress in this area could usher in a new era of electronic materials, integrating the complexities of quantum mechanics with real-world applications that could transform our approach to electronics and information processing.

Hence 2D β-BiAsO_2_ experimentally realized using bismuth oxide (Bi_2_O_3_), arsenic trioxide (As_2_O_3_), silicon dioxide (SiO_2_) or h-BN substrate, solvents (*e.g.*, ethanol, water), precursor solutions (if using a sol–gel process), and high-temperature furnace or chemical vapor deposition (CVD) setup. Synthesizing and characterizing β-BiAsO_2_ as a 2D topological insulator demands advanced growth techniques, such as Molecular Beam Epitaxy (MBE) and Chemical Vapor Deposition (CVD), along with a range of structural, electronic, and magnetic characterization methods. Achieving β-BiAsO_2_ could lead to the development of new topological devices that operate at room temperature; however, precise control over synthesis parameters and substrate interactions is crucial for maintaining its topological characteristics.

## 4 Conclusions

The limited availability of effective quantum spin Hall (QSH) insulators that possess a notable bulk gap presents a significant obstacle to the large-scale implementation of the QSH effect. In this study, first-principles calculations are utilized to explore the stability, electronic characteristics, and topological features of a fully oxygenated bismuth arsenide system. In the absence of spin–orbit coupling (SOC), the valence and conduction bands at the *Γ*-point show a semimetallic behavior. Conversely, once SOC is accounted for, a considerable band gap of 352 meV is produced, allowing for functionality at room temperature. The evaluation of the topological invariant indicates 
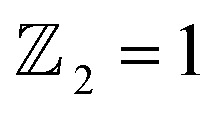
, while topologically protected edge states observed in a Dirac cone at the *Γ*-point affirm the presence of a nontrivial topological state. The epitaxial integration of β-BiAsO_2_ onto a SiO_2_ substrate preserves the band topology of β-BiAsO_2_, demonstrating a significant band gap without SOC alongside a spin lock feature when SOC is present. Furthermore, the fully oxidized surfaces of β-BiAs display a natural resistance to oxidation and degradation, pointing towards a promising pathway for the advancement of room-temperature topological quantum devices.

## Data availability

Band structure of free standing β-BiAs monolayer, optimized structures of Bi–As–O, Bi–As–O and O–Bi–As–O charge densities, Functional structures and Band structures of BiAsX (X = Br_2_, Cl_2_, CH_3_, CN, F_2_, H_2_, I_2_, NH_2_ and O_2_), HSE06, optical properties, for experimental suggestion for further Synthesizing and characterizing β-BiAsO_2_ on a SiO_2_ substrate.

## Conflicts of interest

The authors declare no conflicts of interest.

## Supplementary Material

RA-015-D5RA01911G-s001

RA-015-D5RA01911G-s002
