# Supplementary material for: Exploring a new topological insulator in β-BiAs oxide
Source: RSC Adv. 2025 Apr 28;15(17):13703–11. doi: 10.1039/d5ra01911g (PMC12036511; doi:10.1039/d5ra01911g)
Supplement: RA-015-D5RA01911G-s001 [file RA-015-D5RA01911G-s001.pdf]

 Please do not adjust margins

ARTICLE

Supporting Information

Exploring a New Topological Insulator In β-BiAs Oxide

Tamiru Teshome

Nanotechnology Center of Excellence, Addis Ababa Science and Technology University

College of Natural and Applied Sciences, Department of Mathematics, Physics and Statistics, P. O. Box 16417, Addis Ababa, Ethiopia.

Corresponding authors; E-mail: - tamiru.teshome@aastu.edu.et

Phone: - +251 966253809

ORCID: - 0000-0002-5507-2170

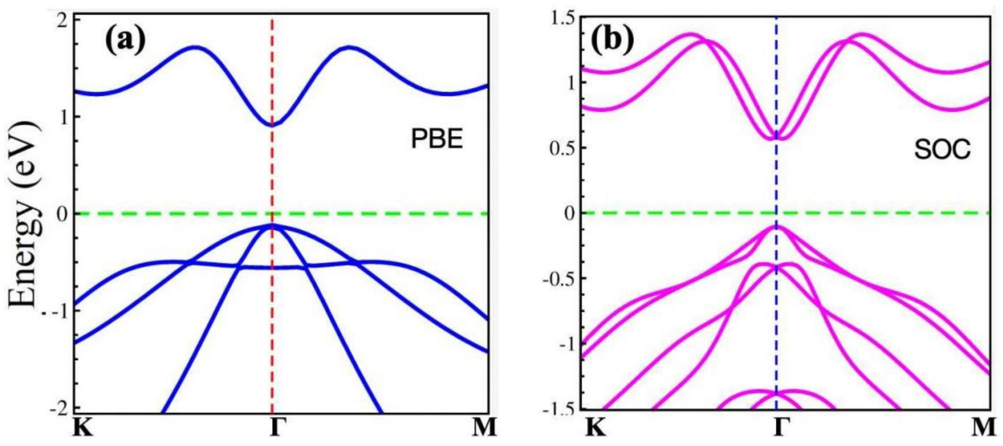

Please do not adjust margins

Figure S1: (a) Electronic band structures of free standing β-BiAs (a) without and (b) With SOC, respectively.

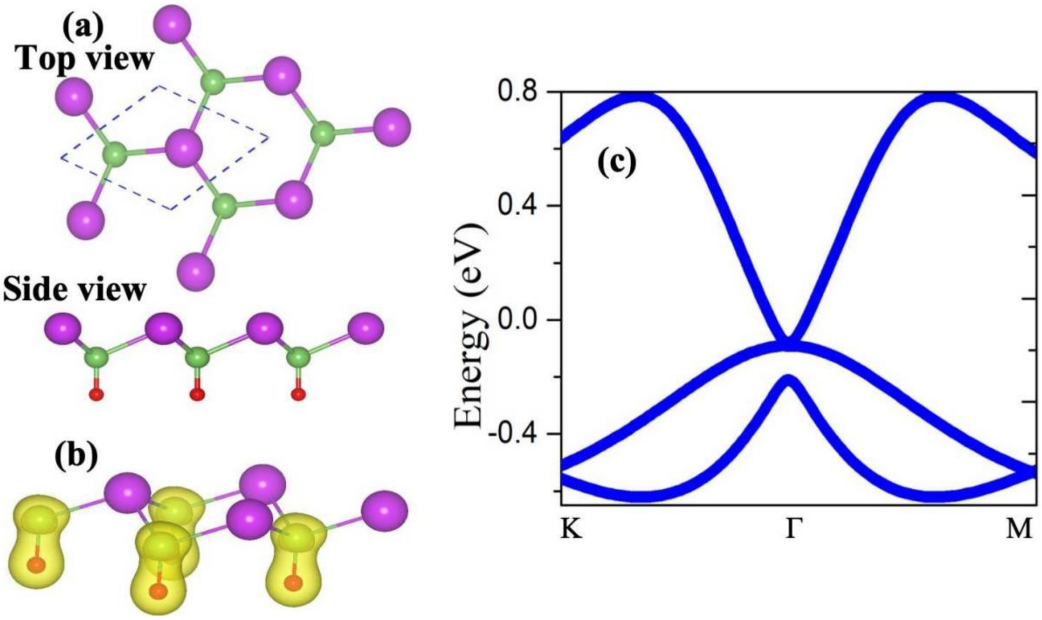

Figure S2: (a) The optimized structure of Bi-As-O top view and side view, (b) charge density and band structure Bi-As-O.

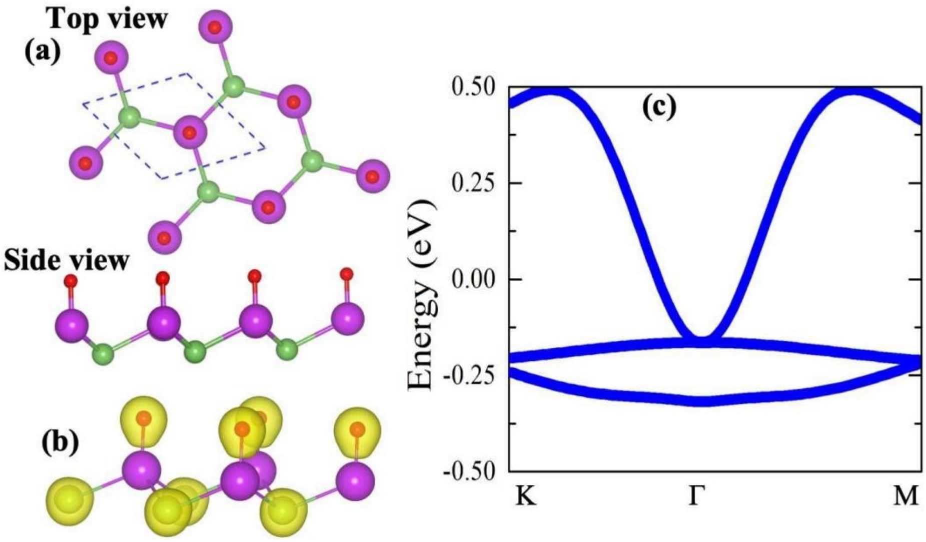

Figure S3: (a) The optimized structure of O-Bi-As top view and side view, (b) charge density and band structure Bi-As-O

 This journal is © The Royal Society of Chemistry 20xx

Please do not adjust margins

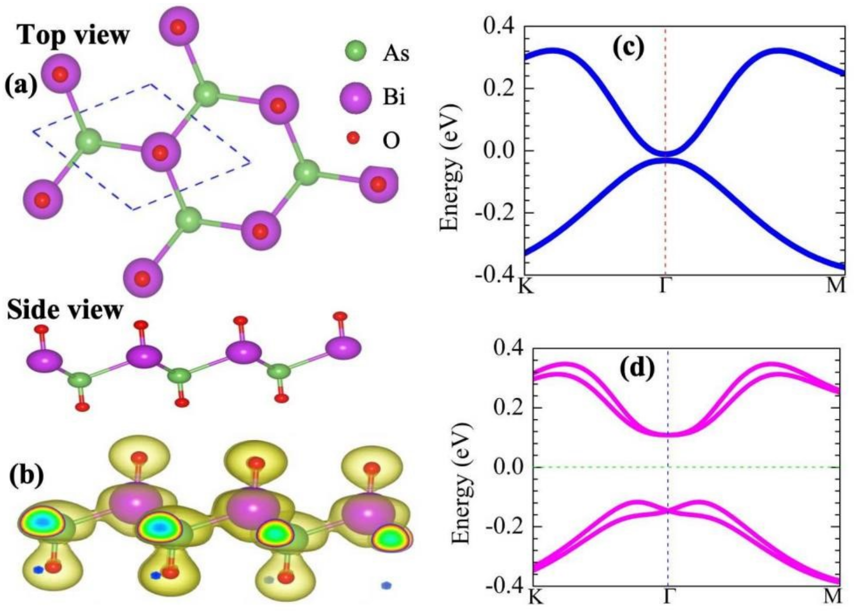

Figure S4: (a) The optimized structure of O-Bi-As-O top view and side view, (b) charge density and (c) O-Bi-As-O band structure without SOC and (d) with SOC effect.

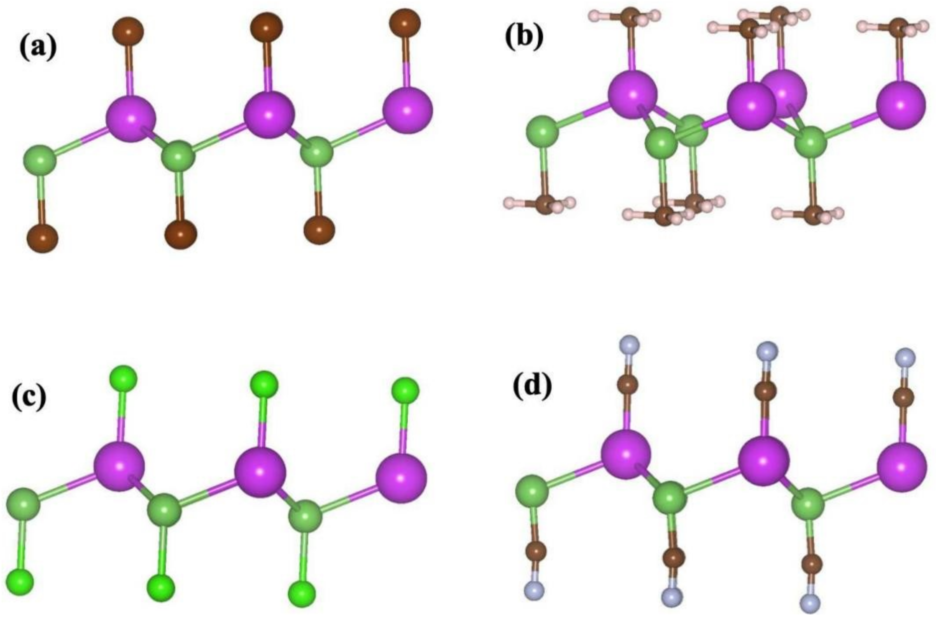

Figure S5: The radical functionality structure of (a) Br-Bi-As-Br, (b) CH3-Bi-As-CH3, (c) Cl-Bi-As-Cl, and CN-Bi-As-CN

Please do not adjust margins

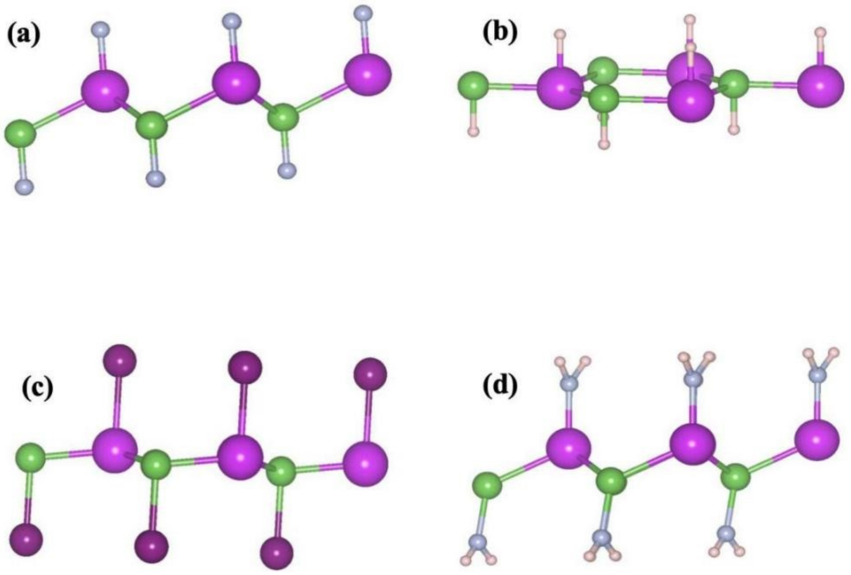

Figure S6: The radical functionality structure of (a) F-Bi-As-F, (b) H-Bi-As-H, (c) I-Bi-As-I, and NH2-Bi-As-NH2

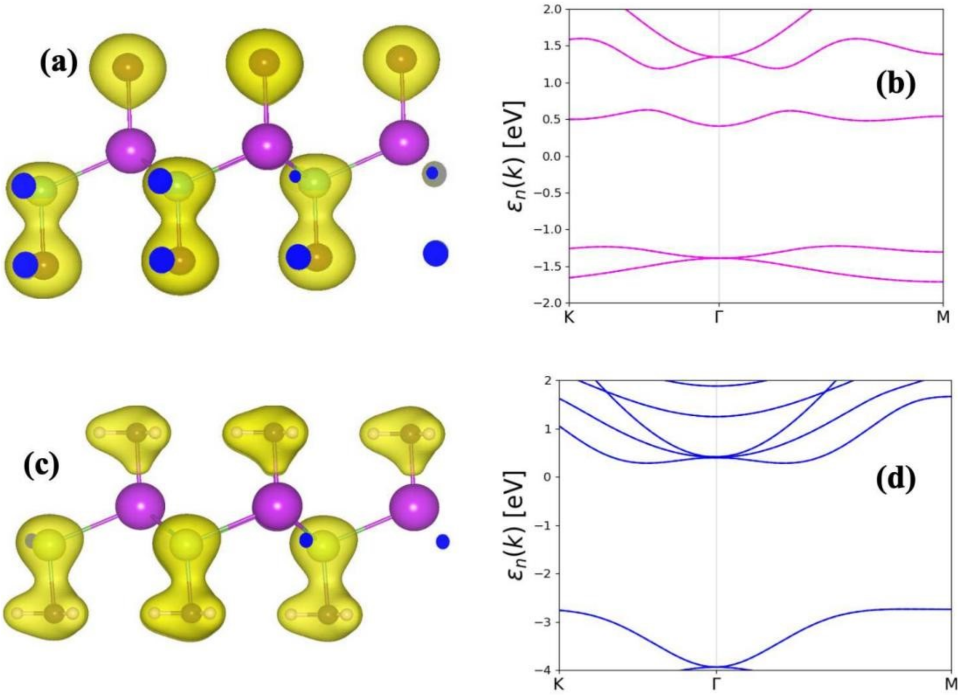

Figure S7: Radical functionality (a,b) charge density and band structure of Br-Bi-As-Br, (c,d) charge density and band structure of CH3-Bi-As-CH3.

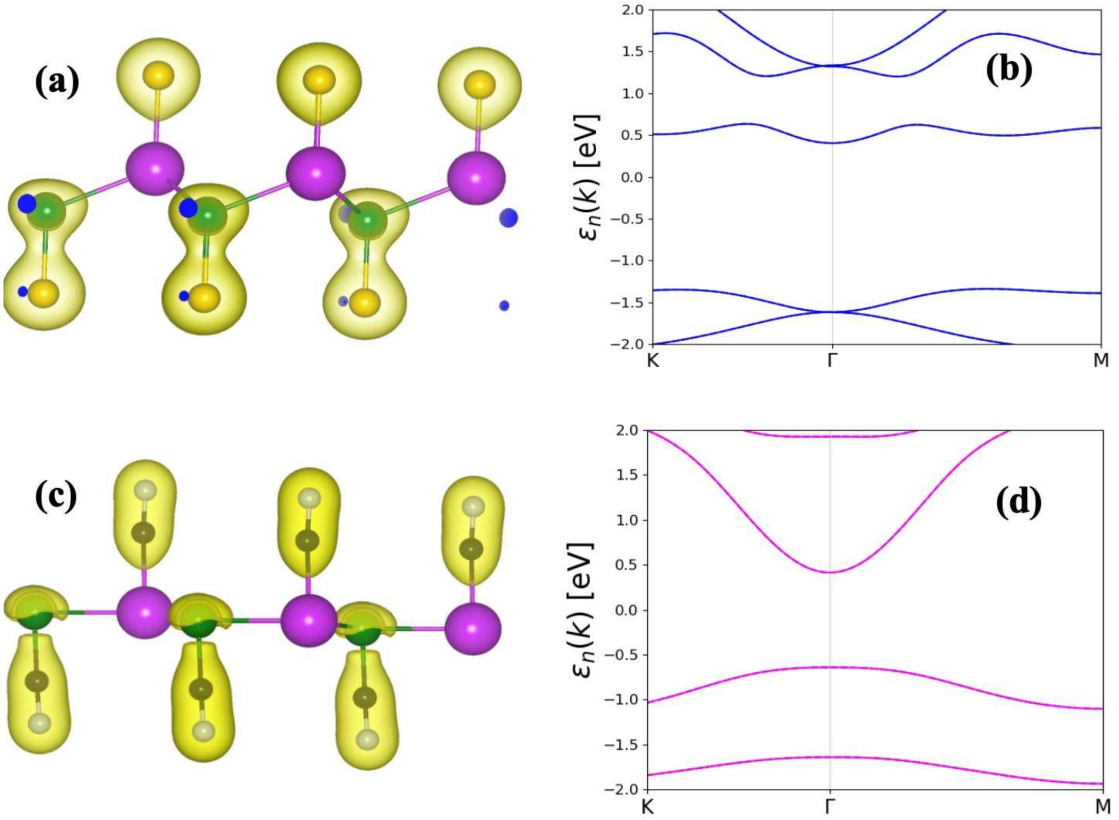

Figure S8: Radical functionalization (a,b) charge density and band structure of Cl-Bi-As-Cl, (c,d) charge density and band structure of CN-Bi-As-CN.

Please do not adjust margins

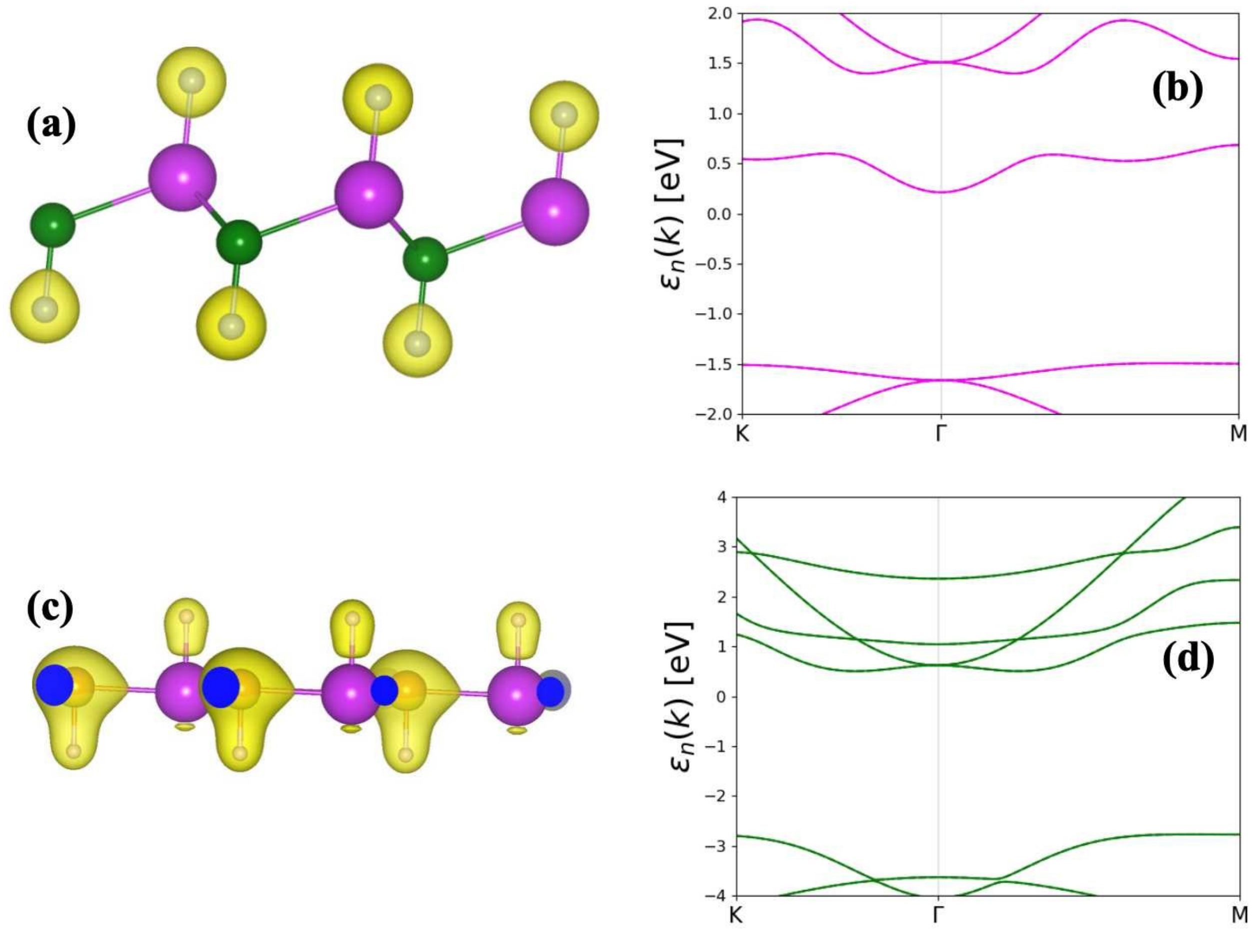

Figure S9: Radical functionality (a,b) charge density and band structure of F-Bi-As-F, (c,d) charge density and band structure of H-Bi-As-H.

 This journal is © The Royal Society of Chemistry 20xx

Please do not adjust margins

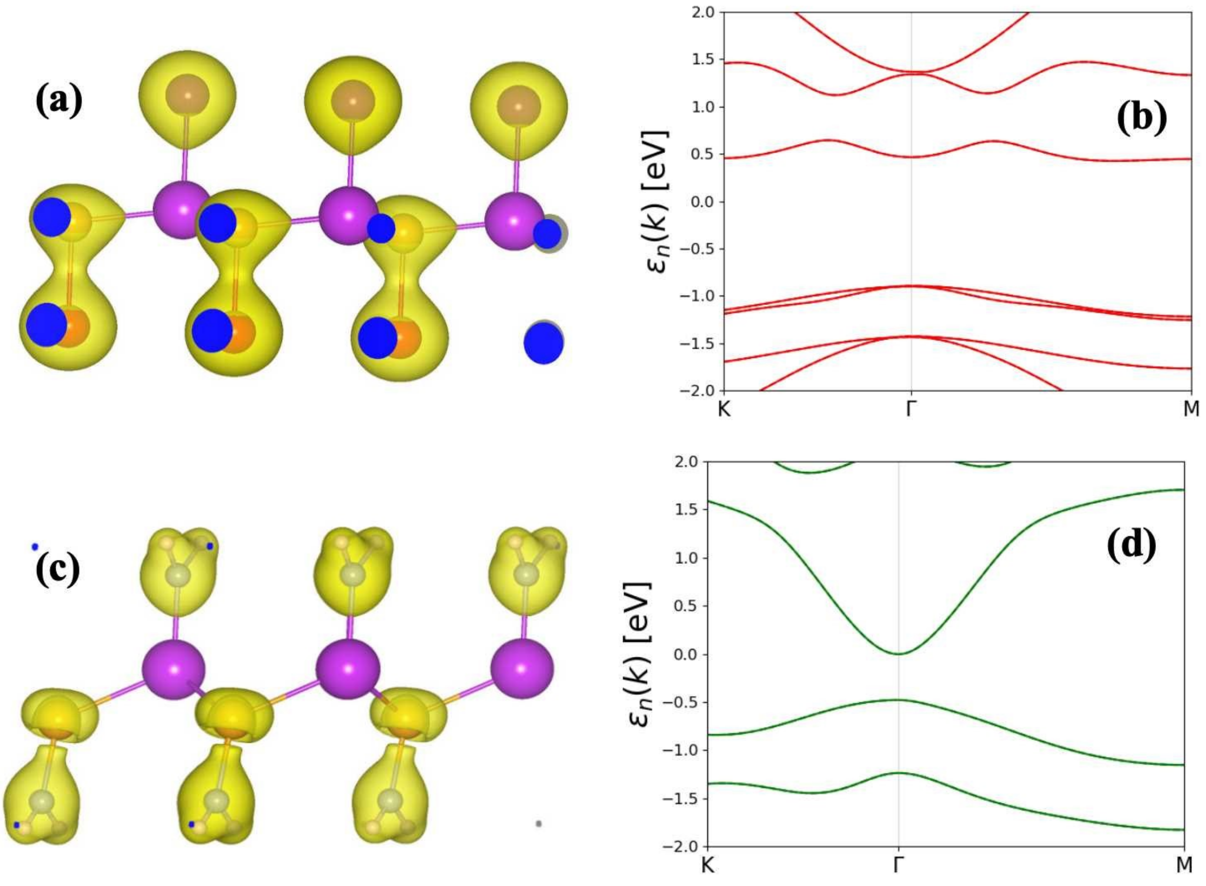

Figure S10: Radical functionality (a,b) charge density and band structure of I-Bi-As-I, (c,d) charge density and band structure of NH2-Bi-As-NH2.

Please do not adjust margins

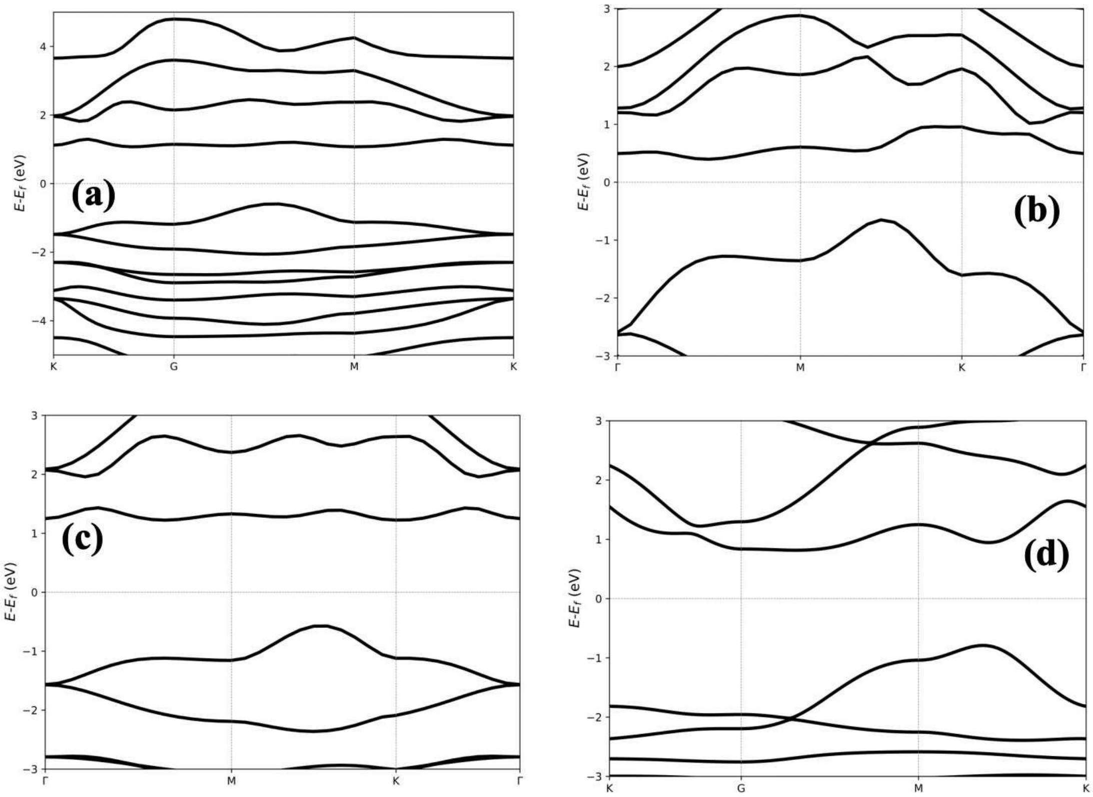

Figure S11: The hybrid functional (HSE06) band structures of (a) Br-Bi-As-Br, (b) CH3-Bi-As-CH3, (c) Cl-Bi-As-Cl, and (d) CN-Bi-As-CN

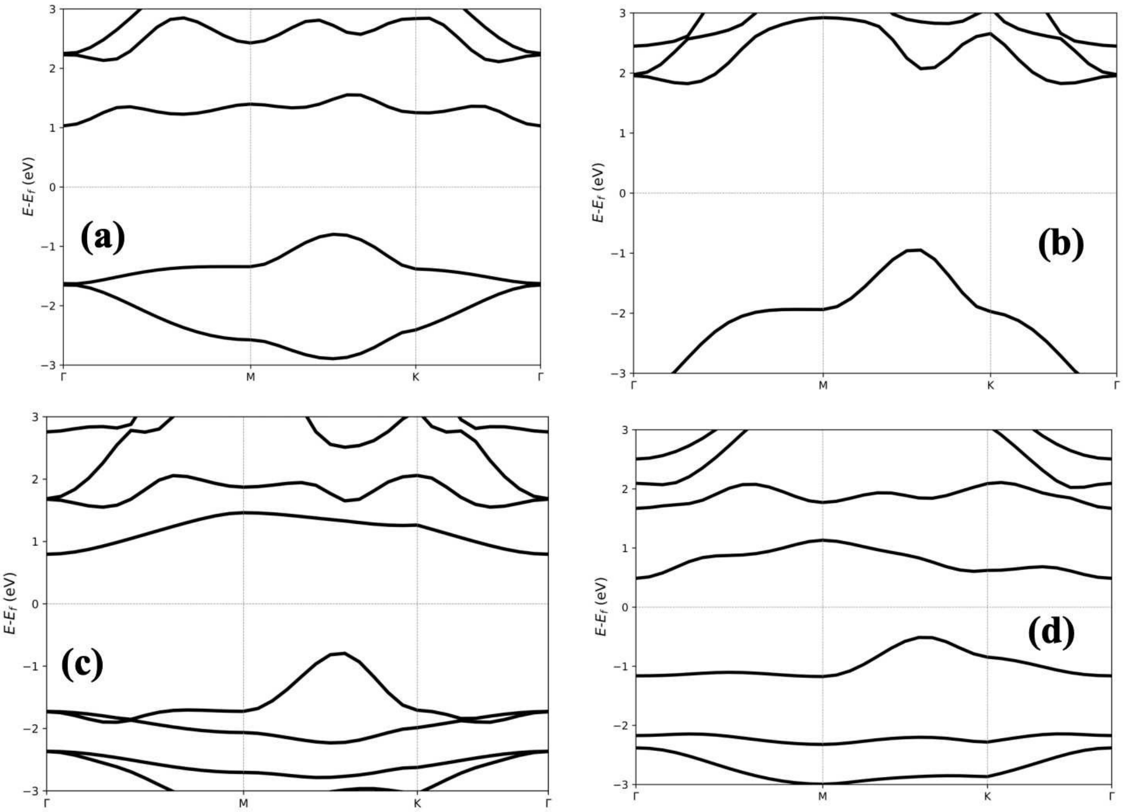

This journal is © The Royal Society of Chemistry 20xx

Please do not adjust margins

Figure S12: The hybrid functional (HSE06) band structures of (a) F-Bi-As-F, (b) H-Bi-As-H, (c) I-Bi-As-I, and NH2-Bi-As-NH2

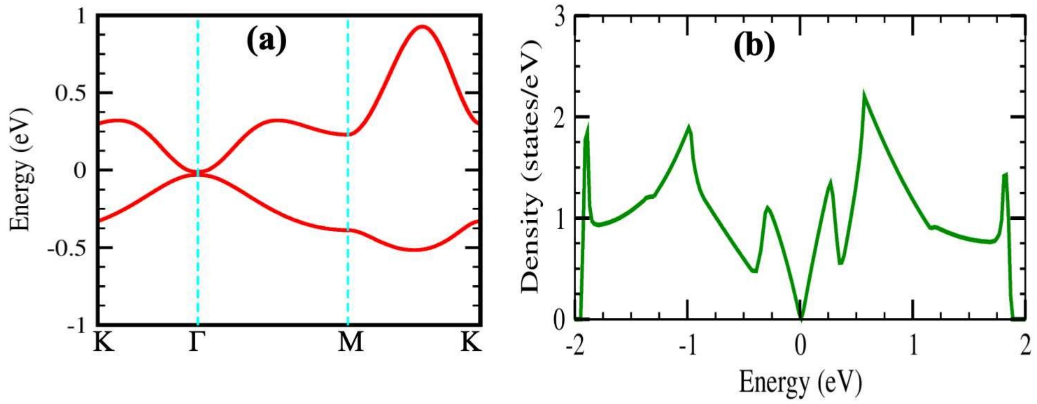

Figure S13: The hybrid functional (HSE06) band structures of (a) O-Bi-As-O, (b) density state

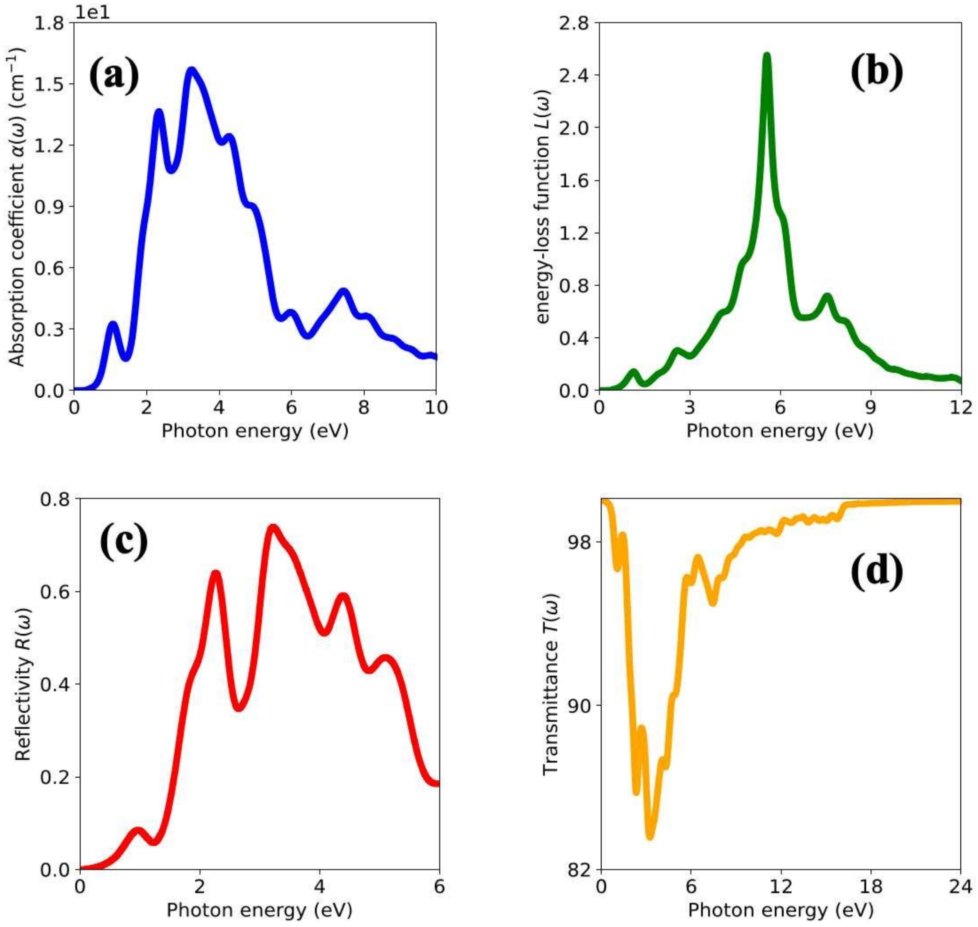

Figure S14: Optical properties of (a) absorption coefficient, (b) energy loss function, (c) reflectivity index, and (d) transmittance of BiAsO2

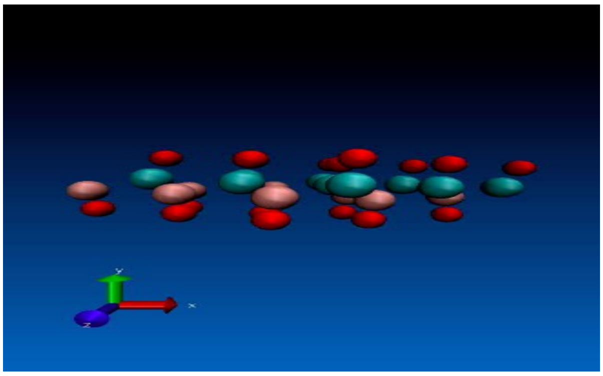

Figure S15: Molecular dynamics simulation at 300K

Suggestion for Experimental:

How to synthesize and characterize β-BiAsO2, a promising 2D TI material

The synthesis and characterization of 2D Topological Insulator (TI) β-BiAsO2 involve advanced material growth techniques and a combination of structural, electronic, and topological characterization methods. Below is a detailed guide on how to synthesize and characterize β-BiAsO2, a promising 2D TI material.

I. Synthesis of β-BiAsO2

Materials

Bismuth oxide (Bi2O3), Arsenic trioxide (As2O3), Silicon dioxide (SiO2) substrate, Solvents (e.g., ethanol, water), Precursor solutions (for sol-gel), High-temperature furnace or CVD setup.

Method:

1. Preparation of Precursors

Dissolve appropriate amounts of Bi2O3 and As2O3 in a suitable solvent (e.g., ethanol or water) to form a homogeneous solution. The molar ratio of Bi to As should be 1:1 to ensure stoichiometric formation of β-BiAsO2.

2. Material Growth Techniques

This journal is © The Royal Society of Chemistry 20xx
Please do not adjust margins

Please do not adjust margins

Molecular Beam Epitaxy (MBE) is a highly controlled technique for growing thin films and 2D materials with atomic precision. For β-BiAsO2, bismuth (Bi), arsenic (As), and oxygen (O) sources are evaporated in an ultra-high vacuum (UHV) chamber and deposited onto a suitable substrate (e.g., h-BN, SiO2, or other insulating substrates). The substrate temperature, deposition rate, and stoichiometry are carefully controlled to ensure the formation of the desired β-BiAsO2 phase. Chemical Vapor Deposition (CVD) is another widely used method for synthesizing 2D materials. Precursors containing Bi, As, and O are introduced into a reaction chamber, where they decompose and react to form β-BiAsO2 on a substrate. The process parameters (temperature, pressure, gas flow rates) are optimized to achieve high-quality, single-crystal β-BiAsO2. Hydrothermal methods can be used to synthesize β-BiAsO2 by reacting Bi and As precursors in an oxygen-rich aqueous solution at high temperatures and pressures. This method is particularly useful for producing bulk crystals, which can later be exfoliated into 2D layers. If β-BiAsO2 bulk crystals are available, mechanical exfoliation (similar to the Scotch tape method used for graphene) can be employed to obtain thin layers. This method is less controllable than MBE or CVD but can produce high-quality 2D flakes for initial studies. The choice of substrate is critical for preserving the topological properties of β-BiAsO2. h-BN (hexagonal boron nitride) is a preferred substrate due to its atomically flat surface, insulating nature, and weak van der Waals interaction with the grown material. Other substrates, such as SiO2/Si or sapphire, can also be used, but their impact on the electronic properties of β-BiAsO2 must be carefully evaluated.

II. Characterization of β-BiAsO2

a) Structural Characterization

Scanning Tunneling Microscopy (STM) offers atomic-resolution images of the surface, enabling the assessment of the crystal structure and quality of the synthesized β-BiAsO2. It can also detect defects, domain boundaries, and other structural irregularities. Transmission Electron Microscopy (TEM) is utilized to analyze the atomic arrangement and thickness of the β-BiAsO2 layers. High-Resolution TEM (HRTEM) can verify the crystalline structure and identify stacking faults or dislocations. X-ray Diffraction (XRD) is used to determine the crystal structure and phase purity of β-BiAsO2, providing insights into lattice parameters and orientation. Raman Spectroscopy identifies specific vibrational modes of β-BiAsO2, aiding in confirming its formation and evaluating its quality, while also revealing any strain or doping effects.

b) Electronic and Topological Characterization

Angle-Resolved Photoemission Spectroscopy (ARPES) is an effective method for investigating the electronic band structure of β-BiAsO2. It can verify the existence of topological surface states and band

Please do not adjust margins

Please do not adjust margins

inversion, which are key characteristics of topological insulators. Electrical transport measurements, including resistivity and the Hall effect, are employed to examine the conductive properties of β-BiAsO2. The presence of edge states can be indicated through non-local transport measurements or studies of the quantum Hall effect. Scanning Tunneling Spectroscopy (STS), which builds on STM, assesses the local density of states (LDOS) and can detect the energy gap and topological surface states. Additionally, optical techniques such as photoluminescence (PL) and absorption spectroscopy provide valuable information about the electronic bandgap and excitonic properties of β-BiAsO2.

c) Magnetic and Spin-Orbit Coupling Characterization

- Spin-Resolved ARPES: Technique can directly measure the spin texture of the surface states, confirming their topological nature.
- Magneto-Transport Measurements: Under applied magnetic fields can reveal the presence of weak anti-localization, a signature of strong spin-orbit coupling (SOC) in topological insulators.

III. Challenges in Synthesis and Characterization

Achieving the correct Bi:As:O ratio is critical for forming the β-BiAsO2 phase. Deviations can lead to the formation of other phases. The choice of substrate can influence the electronic properties of β-BiAsO2. Insulating substrates like h-BN are preferred to minimize interactions. Defects and impurities can disrupt the topological surface states, making high-quality synthesis and careful characterization essential.

The successful synthesis and characterization of β-BiAsO2 on a SiO2 substrate necessitate meticulous preparation of precursors, suitable deposition methods, and comprehensive analysis of the material's properties. Each phase must be fine-tuned according to the specific needs of the application. Sythesizing and characterizing β-BiAsO2 as a 2D topological insulator demands advanced growth techniques, such as Molecular Beam Epitaxy (MBE) and Chemical Vapor Deposition (CVD), along with a range of structural, electronic, and magnetic characterization methods. Achieving β-BiAsO2 could lead to the development of new topological devices that operate at room temperature; however, precise control over synthesis parameters and substrate interactions is crucial for maintaining its topological characteristics.

This journal is © The Royal Society of Chemistry 20xx

Please do not adjust margins
